# Molecular Species Delimitation and Morphology of Aquatic and Sub-Aquatic Bugs (Heteroptera) in Cameroon

**DOI:** 10.1371/journal.pone.0154905

**Published:** 2016-05-05

**Authors:** Solange Meyin A Ebong, Elsa Petit, Philippe Le Gall, Ping-Ping Chen, Nico Nieser, Eric Guilbert, Flobert Njiokou, Laurent Marsollier, Jean-François Guégan, Dominique Pluot-Sigwalt, Sara Eyangoh, Myriam Harry

**Affiliations:** 1 Institut de Recherche pour le Développement, BP 1857, Yaoundé, Cameroon; 2 Service de Mycobactériologie, Centre Pasteur du Cameroun, BP 1274, Yaoundé, Cameroon; 3 UMR EGCE (Évolution, Génomes, Comportement, Écologie), CNRS IRD- Université Paris-Sud, Université Paris-Saclay, Gif-sur-Yvette Cedex, Gif-sur-Yvette, France; 4 UMR MIVEGEC IRD, CNRS, Universités de Montpellier, Centre IRD de Montpellier, Montpellier, France; 5 Inserm Avenir ATOMycA CRCNA Inserm U892 & CNRS U6299, Université et CHU d'Angers, Angers, France; 6 Université de Yaoundé I, Laboratoire de Parasitologie et Ecologie, Faculté des Sciences, Yaoundé, Cameroon; 7 National Museum of Natural History, Naturalis, Leiden, Netherlands; 8 Museum National d’Histoire Naturelle, Département Systématique et Evolution, UMR7205 CNRS/MNHN, Paris, France; University of Veterinary Medicine Hanover, GERMANY

## Abstract

Aquatic and semi-aquatic bugs (Heteroptera) represent a remarkable diversity and a resurging interest has been given to documenting at the species level these insects inhabiting Cameroon in Central Africa due to their potential implication in the transmission of the bacterium *Mycobacterium ulcerans*, the causal agent of Buruli ulcer, an emerging human disease. A survey was carried out over two years in Cameroon. Morphological analyses were done in two steps. A first step consisted in separating the specimens based on broadly shared characters into morphotypes. The specimens were then separated into two independent batches containing each the same representation of each morphotype. One batch (309 specimens) was used by taxonomy experts on aquatic bugs for species level identification and/or to reconcile nymph with their corresponding adult species. The second batch (188 specimens) was used to define species based on the COI DNA sequences (standard sequence used for “DNA barcoding”) and using the Automatic Barcode Gap Discovery (ABGD) method. The first morphological analysis step separated the specimens into 63 different morphotypes (49 adults and 14 nymphs), which were then found to belong to 54 morphological species in the infra-orders Gerromorpha and Nepomorpha based on the species-level morphological identification, and 41–45 putative molecular species according to the gap value retained in the ABGD. Integrating morphology and “DNA barcoding” reconciled all the specimens into 62 aquatic bug species in Cameroon. Generally, we obtained a good congruence between species a priori identified based on morphology from adult morphotypes and molecular putative species. Moreover, molecular identification has allowed the association of 86% of nymphs with adults. This work illustrates the importance of integrative taxonomy.

## Introduction

Aquatic and sub-aquatic true bugs (Heteroptera), comprised in the Leptopodomorpha, the Gerromorpha and the Nepomorpha infra-orders, represent a remarkable species diversity of the aquatic biota with 4,656 species from 326 genera and 20 families found worldwide except in Antarctica [1]. Several surveys of aquatic bugs were carried out in the 1940s in Africa, specifically in West and Central Africa, i.e. the Ivory Coast [2] and Cameroon [3–5]. After this period, the aquatic bugs of West Africa were not studied for decades. But since the 2000s, a resurging interest has grown, documenting the species of aquatic and sub-aquatic bugs inhabiting Cameroon because some of them are suspected to be implicated in the transmission of *Mycobacterium ulcerans*, the causal agent of an emerging human disease named Buruli ulcer [6–11].

The current taxonomy of bugs is mostly based on morphological characters of adults, which are more or less reliable because of their intraspecific variability. Moreover, immature forms are difficult to identify based only on morphology because of the lack of discriminating characters [12]. Therefore, complementary approaches must be developed to address taxonomic issues in Heteroptera. In that regard, Wheeler *et al*. [13] have shown a good congruence between morphological and 18S molecular data to delineate infraorders of Heteroptera but at the species level, more variable sequences than 18S are required. In order to identify taxa which are difficult to separate only on the basis of their morphology, different authors have proposed the “DNA barcoding” which uses a standard region of the mitochondrial gene Cytochrome Oxidase subunit I (COI) [14–16]. Several studies have now established that the COI gene is very useful in insect taxonomy including Hemiptera, especially aphids [17–19], but also true bugs [17].

Initial work [20, 21] reported some limitations using COI-based identification for some Heteropteran groups, but in recent studies, true bug taxa have been identified at both family and species levels, from Asia [17], Korea [22] or Brazil [23].

With the growing implementation of DNA barcoding, it is now possible to not only assign a sample to a pre-existing classification but also to identify unknown species and to decide whether species should be separated or merged using various delimitation methods. Two main classes of methods exist: distance-based methods which consist in clustering sequences in Molecular Operational Units (MOTUs), e.g. the Automatic Barcoding Gap Discovery method (ABGD) [24] and phylogeny-based, such as the Generalized Mixed Yule Coalescent method (GMYC) [25] or the Poisson Tree Processes (PTP) [26].

Here, we integrate both methods based on morphology and molecular data. The ABGD method with COI sequences was applied for the molecular species delimitation. This study aims to contribute to better understand the Cameroon’s biodiversity of aquatic and sub-aquatic bugs putatively involved in the transmission to humans of the environmentally-persistent bacteria *Mycobacterium ulcerans*.

## Materials and Methods

### Sampling of Water Bugs across Cameroon, Africa

**Fig 1:**
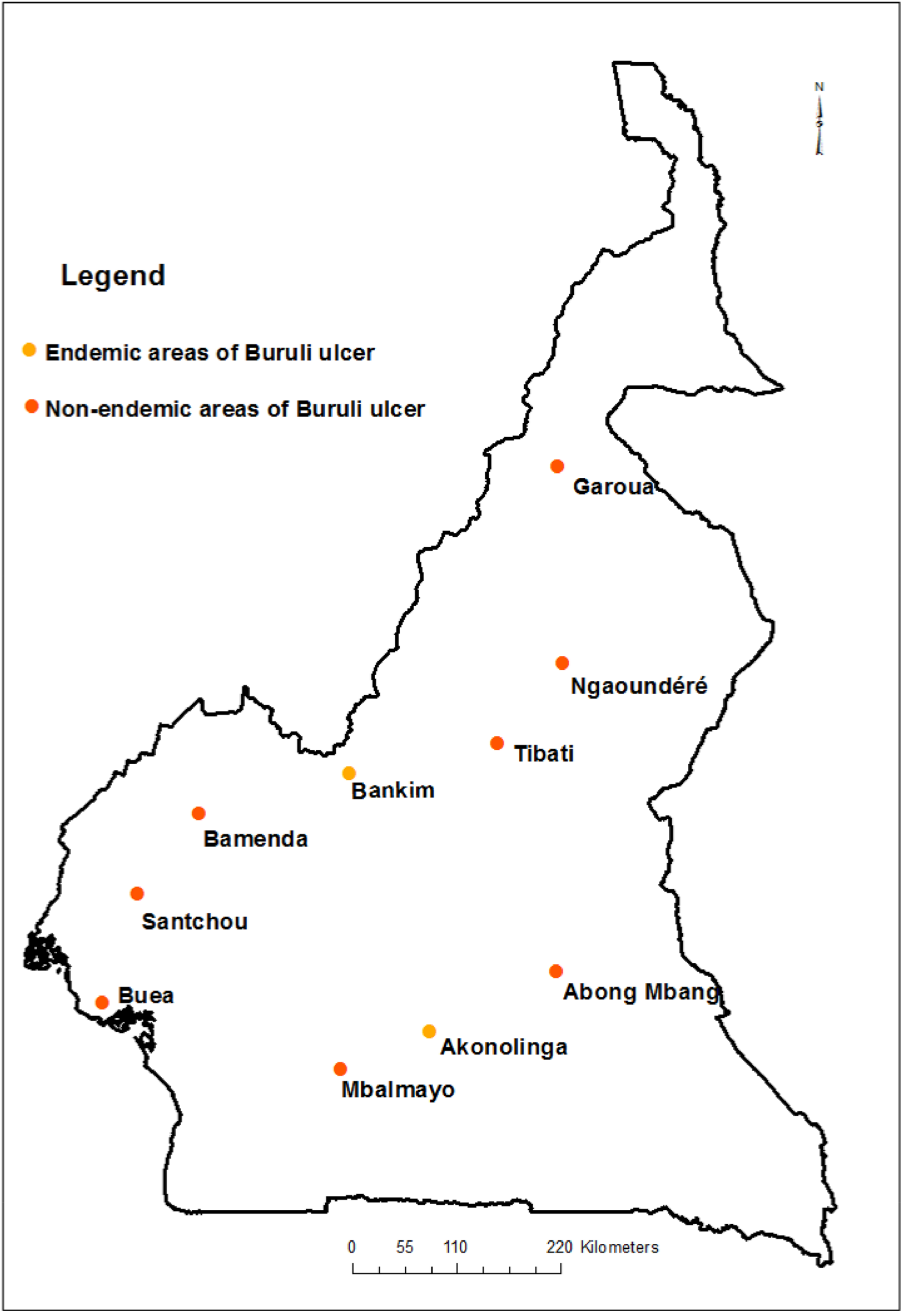
Samples sites of aquatic bugs in Cameroon with information about endemicity of Buruli ulcer. Red-colored points correspond to non-endemic Buruli ulcer zones; yellow-colored points correspond to endemic Buruli ulcer zones.

According to The catch insects’ authorization has been issued by the Director of Wildlife and Protected Areas of the Ministry of Forests and Wildlife which is responsible for the field studies nationwide Decision N° 0859/PCBS/MINFOF/SG/DFAP/SDVEF/SC, insects were collected in 10 locations in Cameroon including two existing endemic zones for Buruli ulcer (Akonolinga and Bankim) and eight non-endemic zones with the same ecological characteristics that the two endemic ones (Mbalmayo, Abong-Mbang, Garoua, Tibati, Ngaoundéré, Bamenda, Buéa and Santchou) (Fig 1). A map (Fig 1) was made from field data collected in different sampling sites using Argis software version 10.2.2. Aquatic and sub-aquatic bugs were collected using two sampling methods: directly in aquatic environment by hauling a metallic dip net (32 × 32 cm and 1 mm in mesh size) within a surface of 1 m^2^ and at different depth levels (down to a depth of 1 m), and indirectly by using light trapping to capture winged imagoes. Aquatic sampling, which included a large variety of streams, rivers, swamps and flooded areas, was performed in triplicate at each survey session, on three consecutive mornings. Light traps were installed twice at each survey session in each site from 6:30PM to 11:00PM. Two survey periods were realized in 2011 (March to June) and 2012–2013 (September 2012 to February 2013) in the endemic (4 times and 6 times respectively at each survey period) and non-endemic zones (2 times at each survey period). After collection, adults and nymphs (L5) were selected, counted, and preserved in 70% ethanol, which was changed weekly, for morphological identification and molecular analyses. The 22,375 specimens collected were first classified by family using the Heteroptera classification given by Schuh and Slater [27] and then identified as morphotypes in each family. For each morphotype, two independent batches were used. One (n = 309) was used for advanced morphological identification at the “Museum National d’Histoire Naturelle” (MNHN) in Paris, France and at the National Museum of Natural History (NMNH) in Leidenin, The Netherlands. And the other (n = 188) was used for molecular identification in the EGCE laboratory at Gif-Sur-Yvette, France.

### Morphological Identification

The 309 specimens were identified down to the species or genus using the pioneer taxonomic work of Poisson on Afrotropical aquatic Heteroptera [2–5,28–33], and other more recent major studies [34–36]. When necessary, several collections (MNHN Paris, NMNH Leiden) were also consulted to compare specimens.

### COI Amplification

A total of 188 specimens were analyzed among which 45 nymphs and 143 adults. About four individuals per morphotype were sampled for DNA sequence analysis. Total DNA was extracted from legs or full body for small insects, using the NucleoSpin Tissue XS [37] according to the manufacturer’s instructions. PCR amplifications were done in 20 μl reaction volumes containing 10 μM of each dNTP (Promega), 10 μM of each primer, 0.5 U of Taq DNA Polymerase (Promega), 1× PCR Buffer (Promega), and DNA extract at about 1/μl. The gene fragments (COI) was amplified using the following pairs of the specific primers LCO 5’-GGTCAACAAATCATAAAGATATTGG-3’ and HCO 5’-TAAACTTCAGGGTGACCAAA AAATCA-3’[38].

PCR always started with a denaturation step of 94°C for five min, followed by 25 cycles comprising denaturation at 94°C, for one min, annealing at 50°C for 1.5 min and elongation at 72°C for one min, and ended with a ten min final elongation at 72°C. PCR products were cleaned by Exosap IT [39], a single-step enzymatic clean up that eliminates unincorporated primers and dNTPs. The COI region was sequenced in the cleaned products.

### Alignments and Phylogenetic Analyses

The cleaned products were then sequenced to 690 bp nucleotides. Multiple alignments were made using Clustal W according to the default settings: full multiple alignments with bootstrap number equal to 1000. Finally, we obtained a data set of 171 COI homologous sequences of 669 bp. We corroborated the (99%) homology of our sequences with COI sequences obtained from BLAST-GenBank and added to our data set some Genbank reference sequences of COI (see Table 1 for accession numbers).

**Table 1:** Accession numbers of sequences used for comparison.

Species name	gi number
*Gerris incognitus*	835444006
*Gerris pingreensis*	835445531
*Gerris insperatus*	835441952
*Limnoporus notabilis*	835436700
*Hydrometra stagnorum*	685164543
*Hydrometra martini*	835440404
*Mesovelia furcata*	685164925
*Rhagovelia obesa*	835427944
*Rhagovelia tenuipes*	381138988
*Aquarius remigoides*	306993637
*Rhagovelia obesa*	835427944
*Microvelia douglasi douglasi*	657639345
*Graphocephala cythura*	227937233

We ran a Maximum Likelihood analysis within MEGA version 5 [40] adding the outgroup *Graphocephala cythura* (Table 1). We used the command “best DNA models” in MEGA, which computes the maximum likelihood fits and selects the best model for 24 different nucleotide substitution models. The best score was obtained for GTR+G+I. The evolutionary history was inferred by using the Maximum Likelihood method based on the Tamura-Nei model. The bootstrap consensus tree inferred from 1000 replicates (Felsenstein, 1985) was taken to represent the evolutionary history of the taxa analyzed. Initial tree(s) for the heuristic search were obtained automatically as follows. When the number of common sites was < 100 or less than one fourth of the total number of sites, the maximum parsimony method was used; otherwise BIONJ method with MCL distance matrix was used. A discrete Gamma distribution was used to model evolutionary rate differences among sites (5 categories (+G, parameter = 0.6035)). The rate variation model allowed for some sites to be evolutionarily invariable ([+I], 42.9957% sites). The trees were drawn to scale, with branch lengths measured in the number of substitutions per site. All ambiguous positions were removed for each sequence pair.

### Molecular-Based Species Delimitation

Molecular putative species limits were explored with the Automatic Barcode Gap Discovery (ABGD) [24]. This method uses the distribution of pairwise genetic distances to separate samples into putative species. The distribution of pairwise distances is bimodal with intra-specific variation and inter-specific variation separated by the barcode gap that is used as a threshold to delimit species. Alignments were uploaded at http://www.abi.snv.jussieu.fr/public/abgd/abgd.html and ABGD was run with the default settings (Pmin = 0.001, Pmax = 0.1, Steps = 10, X (relative gap width) = 1, Nb bins = 20) and with K2P distances. The data can be partitioned into finer and finer partitions until there is no further partitioning. Tree different partitions were used in this study: p1 (p = 0.0359); p2 (p = 0.0046); p3 (p = 0.0028).

## Results

### Morphological Identification

Based on morphology, the 309 specimens of aquatic and sub-aquatic bugs studied were firstly grouped into 49 adult morphotypes. After the advanced morphological analysis comparing specimens to the aquatic and sub-aquatic bugs collections preserved in the Museums (MNHN, France; NMNH, The Netherlands), these morphotypes were dispatched in 54 species belonging to 11 different families (Tables 2 and 3). Indeed, each of ten morphotypes were further separated in two distinct species: Mor13 separated into *Macrocoris flavicollis flavicollis* and *Macrocoris laticollis laticollis*, Mor16 separated into *Laccotrephes dilatatus* and *Laccotrephes fabricii*, Mor17 separated into *Laccotrephes calcaratus* and *Laccotrephes armatus*, Mor18 separated into *Laccotrephes latimanus* and *Laccotrephes* sp., Mor21 separated into *Anisops (Micranisops) apicalis* and *Anisops (Micranisops) parvulus*, Mor25 separated into *Anisops (s*. *str*.*) sardeus* and *Anisops* sp., Mor31 separated into *Limnogonus (s*. *str*.*) cereiventris* and *Limnogonus (Limnogoïdes) poissoni*, Mor33 separated into *Neogerris severini* and *Gerris swakopensis*, Mor38 separated into *Hydrometra* sp.1 and *Hydrometra huntchinsoni* and Mor39 separated into *Hydrometra albolineata* and *Hydrometra* sp.2 (Table 2). And in five cases, two morphotypes were merged in only one species: Mor23-25 merged into *Anisops sardeus*, Mor26-27 into *Enithares glauca*, Mor44-48 into *Rhagovelia* sp. 1, Mor40-41 into *Mesovelia* sp., Mor45-46 into *Rhagovelia* sp.2, and Mor30-49 into *Hebrus* sp. (Table 2). Noticeably, for 14 specimens, no species name could be assigned.

**Table 2:** Relationships between adult morphotypes and species defined by morphological analysis in the Nepomorpha infraorder.

Families	MorAd^1^	Loc^2^	N/ sex^3^	Species names defined by morphological analysis
**Belostomatidae**	^a^Mor1^b^	AM, A	5♀, 2♂	*Appasus grassei grassei* (Poisson, 1937)
**Belostomatidae**	Mor2	A, T	2♀, 2♂	*Appasus nepoides* (Fabricius, 1803)
**Belostomatidae**	Mor3	AM, B	4♀, 2♂	*Appasus procerus procerus* (Gerstaecker, 1873)
**Belostomatidae**	Mor4	AM, B	2♀, 3♂	*Appasus ampliatus* Bergroth, 1890
**Belostomatidae**	Mor5	G, M	2♂	*Limnogeton fieberi* Mayr, 1953
**Belostomatidae**	Mor6	N	1♀, 1♂	*Hydrocyrius nanus* Montandon, 1907
**Belostomatidae**	Mor7	G, A	1♀, 3♂	*Hydrocyrius colombiae colombiae* Spinola, 1852
**Belostomatidae**	Mor8	AM, G	1♀, 4♂	***Lethocerus cordofanus* Mayr, 1953**
**Corixidae**	Mor9	B	6♀, 5♂	*Sigara (Tropocorixa) camerounensis* Poisson, 1941
**Micronectidae**	Mor10	G, N	5♀, 14♂	*Micronecta* sp. 1
**Micronectidae**	Mor11	B	2♀	*Micronecta* sp. *2*
**Naucoridae**	Mor12	N	2♀, 2♂	*Naucoris obsuratus obsuratus* Montandon, 1913
**Naucoridae**	Mor13	B, N, T	5♂	*Macrocoris flavicollis flavicollis* Signoret, 1861
**Naucoridae**	Mor13	A-M	2♂	***Macrocoris laticollis laticollis* Montandon, 1913**
**Naucoridae**	Mor14	B	1♂	*Laccocoris discus* Poisson, 1949
**Naucoridae**	Mor15	BU	1♀	*Laccocoris limigenis* Stål 1865
**Nepidae**	Mor16	B	1♀	*Laccotrephes dilatatus* Montandon, 1895
**Nepidae**	Mor16	BA, M	1♀, 3♂	*Laccotrephes fabricii* Stål, 1968
**Nepidae**	Mor17	AM, M	2♀, 2♂	*Laccotrephes calcaratus* Montandon, 1898
**Nepidae**	Mor17	BA	1♀, 1♂	***Laccotrephes armatus* Montandon, 1898**
**Nepidae**	Mor18	B, G	1♀, 1♂	*Laccotrephes latimanus* Montandon, 1909
**Nepidae**	Mor18	AM, B	1♀, 1♂	***Laccotrephes* sp.**
**Nepidae**	Mor19	A	2♀, 1♂	*Ranatra bottegoi* Montandon, 1903
**Nepidae**	Mor20	B	1♀	*Ranatra (Capensis) congoensis* Poisson, 1949
**Notonectidae**	Mor21	AM, BA	7♀, 3♂	***Anisops (Micranisops) apicalis* Stål, 1855**
**Notonectidae**	Mor21	A	5♀, 4♂	*Anisops (Micranisops) parvulus* Brooks, 1952
**Notonectidae**	Mor22	A, BA	2♀, 3♂	*Anisops (s*. *str*.*) kampalensis* Hutchinson, 1928
**Notonectidae**	Mor24	G	11♀, 8♂	*Anisops (s*. *str*.*) jaczewskii* Hutchinson, 1928
**Notonectidae**	Mor23	A	4♂	*Anisops (s*. *str*.*) sardeus* Herrich-Schaeffer, 1849
**Notonectidae**	Mor25	BA	7♀	*Anisops (s*. *str*.*) sardeus* Herrich-Schaeffer, 1849
**Notonectidae**	Mor25	A, T	3♀	***Anisops* sp.**
**Notonectidae**	Mor26	B	6♀	*Enithares glauca* Bolivar, 1879
**Notonectidae**	Mor27	B	2♂	*Enithares glauca* Bolivar, 1879
**Notonectidae**	Mor28	AM, A	10♀, 4♂	*Enithares sobria* Stål, 1855
**Notonectidae**	Mor29	AM	3♀, 1♂	*Nychia marshalli* (Scott, 1872)
**Notonectidae**	MorL14	M	3♀, 1♂	*Neonychia congoensis congoensis* Hungerford, 1946

^**1**^ Adult Morphotypes: ^**a**^ Morphotype code, ^**b**^ Morphotype descending number

^**2**^ Sampling sites: G = Garoua; M = Mbalmayo; A = Akonolinga; S = Santchou; N = Ngaoundére; AM = Abong-Mbang; T = Tibati; B = Bamenda; Ba = Bankim; Bu = Buea.

^**3**^ Specimens number per sex: ♀ = female, ♂ = male

Bold: absent in the molecular species

**Table 3:** Relationships between adult morphotypes and species defined by morphological analysis in the Gerromorpha infraorder.

Family	MorAd^1^	Loc^2^	N/ sex^3^	Species name defined by morphological analysis
Gerridae	^a^Mor31^b^	G, M	12♀, 2♂	*Limnogonus (s*. *str*.*) cereiventris* Signoret, 1862
Gerridae	Mor31	A, S	4♀, 1♂	*Limnogonus (Limnogoïdes) poissoni* Andersen, 1973
Gerridae	Mor32	N	10♀	*Limnogonus (Limnogoides)* sp.
Gerridae	Mor33	A	3♀, 1♂	***Neogerris severini* Kirkaldy, 1900**
Gerridae	Mor33	AM	2♀, 1♂	***Gerris swakopensis* Stål, 1858**
Gerridae	Mor34	B	3♀	*Eurymetra* sp.
Gerridae	Mor35	N	3♀	*Limnogonus (Limnogoïdes) hypoleucus* Gerstaecker, 1873
Gerridae	Mor36	AM, A	2♀, 2♂	*Limnogonus (Limnogoïdes) intermedius* Poisson, 1941
Gerridae	Mor37	M	1♂	*Rhagadotarsus (Caprivia) hutchinsoni* China, 1931
Hydrometridae	Mor38	A, BA	3♀	*Hydrometra* sp.1
Hydrometridae	Mor38	AM, M	2♀, 2♂	*Hydrometra huntchinsoni* Hungerford & Evans, 1934
Hydrometridae	Mor39	BA, M	1♂	*Hydrometra albolineata* Reuter, 1882
Hydrometridae	Mor39	G	2♀	***Hydrometra* sp.2**
Mesoveliidae	Mor40	A, BA	5♂	*Mesovelia* sp.
Mesoveliidae	Mor41	G, T	4♀, 1♂	*Mesovelia* sp.
Mesoveliidae	Mor42	M, N	7♂, 10♀	*Mesovelia vigittigera* Horvath, 1895
Veliidae	Mor43	AM, A	3♀, 5♂	*Angilia* sp.
Veliidae	Mor44	AM, B	1♀, 2♂	*Rhagovelia* sp.1
Veliidae	Mor48	AM	1♂	*Rhagovelia* sp. 1
Veliidae	Mor45	T	3♀	*Rhagovelia* sp. 2
Veliidae	Mor46	G, N	11♀, 7♂	*Rhagovelia* sp. 2
Veliidae	Mor47	S	4♀, 1♂	*Rhagovelia* sp. 3
Hebridae	Mor49	A	2♀	*Hebrus* sp.
Hebridae	Mor30	A	2♀	*Hebrus* sp

^**1**^ Adult Morphotypes: ^**a**^ Morphotype code, ^**b**^ Morphotype descending number

^**2**^ Sampling sites: G = Garoua; M = Mbalmayo; A = Akonolinga; S = Santchou; N = Ngaoundére; AM = Abong-Mbang; T = Tibati; B = Bamenda; Ba = Bankim; Bu = Buea.

^**3**^ Specimens number per sex: ♀ = female, ♂ = male

Bold: absent in the molecular species

In addition, MorL14 initially designated as a nymphal morphotype was identified as an adult of the species *Neonychia congoensis congoensis* Hungerford, 1946. For all remaining nymphal morphotypes it was impossible to attribute a specific name based on the morphology.

### Molecular-Based Species Delimitation

Using the ABGD method, we explored the limits between the different species using the COI sequence from 188 specimens including 49 adult and 14 nymphal morphotypes. The COI amplification failed for some adult morphotypes (Mor8, Mor18, Mor25, Mor33), certainly due to DNA degradation. The molecular data set obtained for the 45 adult morphotypes were partitioned into 41 or 45 putative molecular species according to the gap value retained corresponding to distance values of 0.0359 and 0.0028, respectively (Table 4), (S1 and S2 Figs). Three morphotypes (Mor28, 36, 42) were each split into two molecular species. If the partition 3 is considered, two additional morphotypes Mor7 and Mor38 would also each split into 2 molecular species (Table 4). In five cases, two or three morphotypes were merged into one molecular species (Mor26-27, Mor31-32; Mor40-41-42, Mor44-48, Mor45-46, Mor49-30) (Table 4).

**Table 4:** Putative molecular species and morphological species identifying a posteriori for adult morphotypes according to families.

Family	Mor^1^	ABGD	ABGD	ABGD	Associated species names defined a posteriori by morphological
		p1^2^	p2^3^	p3^4^	
Belostomatidae	Mor1	B1(4)			*Appasus grassei grassei* (Poisson, 1937)
Belostomatidae	Mor2	B3(8)			*Appasus nepoides* (Fabricius, 1803)
Belostomatidae	Mor3	B4(4)			*Appasus procerus procerus* (Gerstaecker, 1873)
Belostomatidae	Mor4	B5(3)			*Appasus ampliatus* (Bergroth, 1890)
Belostomatidae	Mor5	B6(3)			*Limnogeton fieberi* (Mayr, 1953)
Belostomatidae	Mor6	B7(2)			*Hydrocyrius nanus* (Montandon, 1907)
Belostomatidae	Mor7	B8(4)		B8(3)	*Hydrocyrius colombiae colombiae* (Spinola, 1852)
Belostomatidae	Mor7	B8(4)		B2(1)	**No morphological species associated**
Corixidae	Mor9	C1(4)			*Sigara (Tropocorixa)* sp.
Micronectidae	Mor10	C2(4)			*Micronecta* sp. 1
Micronectidae	Mor11	C3(3)			*Micronecta* sp. 2
Naucoridae	Mor12	Na1(4)			*Naucoris obsuratus obsuratus* Montandon, 1913
Naucoridae	Mor13	Na2(4)			*Macrocoris flavicollis flavicollis* Signoret, 1861
Naucoridae	Mor14	Na3(4)			*Laccocoris discus* Poisson, 1949
Naucoridae	Mor15	Na4(2)			*Laccocoris limigenis* Stål, 1865
Nepidae	Mor16	Nm1(4)			*Laccotrephes fabricii* Stål, 1968
Nepidae	Mor17	Nm2(1)			*Laccotrephes calcaratus* Montandon, 1898
Nepidae	Mor19	Nr1(2)			*Ranatra bottegoi* Montandon, 1903
Nepidae	Mor20	Nr2(2)			*Ranatra (Capensis) congoensis* Poisson, 1949
Nepidae	Mor21	No1(4)			*Anisops (Micranisops) parvulus* Brooks, 1952
Notonectidae	Mor22	No2(3)			*Anisops (s*. *str*.*) kampalensis* Hutchinson, 1928
Notonectidae	Mor23	No3(2)			*Anisops (s*. *str*.*) sardeus* Herrich-Schaeffer, 1849
Notonectidae	Mor24	No4(2)			*Anisops (s*. *str*.*) jaczewskii* Hutchinson, 1928
Notonectidae	Mor26	No8(3)			*Enithares glauca* Bolivar, 1879
Notonectidae	Mor27	No8(1)			*Enithares glauca* Bolivar, 1879
Notonectidae	Mor28	No5(1)			**No morphological species associated**
Notonectidae	Mor28	No6(3)			*Enithares sobria* Stål, 1855
Notonectidae	Mor29	No7(4)			*Nychia marshalli* (Scott, 1872)
Gerridae	Mor31	Ge1(2)		Ge1(1)	*Limnogonus (s*. *str*.*) cereiventris* Signoret, 1862
Gerridae	Mor31	Ge2(2			*Limnogonus (Limnogoïdes)* sp.
Gerridae	Mor32	Ge2(2)			*Limnogonus (Limnogoïdes)* sp.
Gerridae	Mor34	Ge5(3)			*Eurymetra* sp.
Gerridae	Mor35	Ge6(4)			*Limnogonus (Limnogoïdes) hypoleucus* Gerstaecker, 1873
Gerridae	Mor36	Ge4(1)			**No morphological species associated**
Gerridae	Mor36	Ge3(3)			*Limnogonus (Limnogoides) intermidus* Poisson, 1641
Gerridae	Mor37	Ge7(2)			*Rhagadotarsus (Caprivia) hutchinsoni* China, 1931
Hydrometridae	Mor38	Hy1(2)	Hy1(1)		*Hydrometra huntchinsoni* Hungerford & Evans, 1934
Hydrometridae	Mor38	Hy1(2)	Hy2(1)		*Hydrometra* sp.
Hydrometridae	Mor39	Hy1(4)	Hy3(4)		*Hydrometra albolineata* Reuter, 1882
Mesoveliidae	Mor40	Me1(3)			*Mesovelia* sp
Mesoveliidae	Mor41	Me1(3)			*Mesovelia* sp
Mesoveliidae	Mor42	Me1(4)			*Mesovelia vigittigera* Horvath, 1895
Veliidae	Mor43	Ve1(3)			*Angilia* sp.
Veliidae	Mor44	Ve2(5)			*Rhagovelia* sp. 1
Veliidae	Mor48	Ve2(4)			*Rhagovelia* sp. 1
Veliidae	Mor45	Ve3(1)			*Rhagovelia* sp. 2
Veliidae	Mor46	Ve3(1)			*Rhagovelia* sp. 2
Veliidae	Mor47	Ve4(3)			*Rhagovelia* sp. 3
Hebridae	Mor49	He1(2)			*Hebrus* sp.
Hebridae	Mor30	He1(2)			*Hebrus* sp.

^1^ Mor: Morphotype code

^2^ ABGD: ABGD putative species with the partition 1 (p = 0.0359)

^3^ ABGD: ABGD putative species with the partition 2 (p = 0.0046)

^4^ ABGD: ABGD putative species with the partition 3 (p = 0.0028)

The number of specimens examined is given in parentheses.

Table 5 shows the assignment of nymph molecular species to adult molecular species and the *a posteriori* species names associated to the corresponding adult determined based on morphology. According to the gap-value used, 11 (p = 0.0359) to 17 (p = 0.0028) molecular species could be recognized for the nymph morphotypes (Table 5). If we considered the partition 3 (p = 0.0028), out of the 14 *a priori* morphological morphotypes, eight could be assigned to a single molecular species (Table 5). The morphotypes, MorL2, MorL4, and MorL8, were split into two or three molecular species (Table 5). Two nymphal morphotypes and MorL10 and MorL11 were merged into one molecular species (No6) (Table 5). The molecular study of the nymphs sample allowed the association of nymphs with adults for 11 species but not for four species (Table 5).

**Table 5:** Assignments of nymph morphotypes to adult morphotypes determined by molecular identification and *a posteriori* species names associated to the corresponding adult as determined based on morphology (Tables 1 and 2).

Family	MorL^1^	ABGD p1^2^	ABGD P2^3^	ABGD P3^4^	MolAd^5^	Morphological species determined *a posteriori*
Belostomatidae	MorL1	ML1(4)			B3	*Appasus nepoides* (Fabricius, 1803)
Belostomatidae	MorL2	ML2(4)		ML2(2)	B8	*Hydrocyrius colombiae colombiae* Spinola, 1852
Belostomatidae	MorL2	ML2(4)		ML3(1)	**No adult**	**No morphological species associated**
Belostomatidae	MorL2	ML2(4)		ML4(1)	**No adult**	**No morphological species associated**
Belostomatidae	MorL3	ML5 (4)			B6	*Limnogeton fieberi* Mayr, 1953
Belostomatidae	MorL4	ML6(3)	ML6(3)		B5	*Appasus ampliatus* Bergroth, 1890
Belostomatidae	MorL4	ML7(1)	ML7(1)		B2	**No morphological species associated**
Naucoridae	MorL5	ML8(4)			Na2	*Macrocoris flavicollis flavicollis* Signoret, 1861
Nepidae	MorL6	ML9 (1)			Nm1	*Laccotrephes fabricii* Stål, 1968
Nepidae	MorL7	ML10(3)			Nm2	*Laccotrephes calcaratus* Montandon, 1898
Notonectidae	MorL8			ML12(1)	**No adult**	**No morphological species associated**
Notonectidae	MorL8	ML11(2)		ML13(1)	**No5**	**No morphological species associated**
Notonectidae	MorL9	ML11(1)				
Notonectidae	MorL10	ML14(1)			No6	*Enithares sobria* Stål, 1855
Notonectidae	MorL11	ML14(1)			No6	*Enithares sobria* Stål, 1855
Notonectidae	MorL12	ML15(4)			No1	*Anisops (Micranisops) parvulus* Brooks, 1952
Notonectidae	MorL13	ML16(4)			No7	*Nychia marshalli* (Scott, 1872)
Mesoveliidae	MorL14	ML17(2)			**No adult**	**No morphological species associated**

^1^ MorL: Nymph morphotype code

^2^ ABGD: ABGD putative species with the partition 1 (p = 0.0359)

^3^ ABGD: ABGD putative species with the partition 2 (p = 0.0046)

^4^ ABGD: ABGD putative species with the partition 3 (p = 0.0028)

^5^ MolAd: Adult molecular putative species code

The number of specimens examined is given in parentheses.

### Morphological and Molecular Aquatic Bugs Species Biodiversity in Cameroon

For the first set of samples (based on morphological criteria), 54 species were determined and for the second set (based on molecular criteria), 52–62 species were putatively delimited using COI molecular marker. Nine species were lacking from the molecular sampling and if we consider the partition p3, 8 putative molecular species have no morphological species associated with them (Tables 4 and 5). But, for the adults, we observed a good congruence between the morphological and the molecular study for the determination of the *a priori* morphotypes. In some cases, the *a priori* determination of morphotypes lead to recognize two species while only one was assessed by the two methods as for two cases in Veliidae (Mor44-48, Mor45-46), Notonectidae (Mor26-27) and Hebridae (Mor30-49). By contrast, two species were recognized by the two methods for a single *a priori* morphological species in Gerridae (Mor31, Mor33), Hydrometridae (Mor38). However, some discrepancies are noticed for Gerridae (Mor31-32) and Mesoveliidae (Mor40-41-42).

If we validate all the morphological species and the molecular species for which no morphological species was associated, the combination of the two dataset (morphological and molecular) yield a total of 62 aquatic bugs species in Cameroon.

The species biodiversity of aquatic bugs varies among identified families. The most diversified families are Notonectidae (13 species), Belostomatidae (11 species), Gerridae (10 species) and Nepidae (8 species). Other families with intermediate diversity are: Micronectidae (2 species), Mesoveliidae (3 species), Hydrometridae and Veliidae (4 species for each family) and Naucoridae (5 species). Corixidae and Hebridae are the least diversified families with only one identified species each (Table 6).

**Table 6:** Checklist of aquatic bugs reported from Cameroon.

Infraorder	Family	Previously reported from Cameroon		Present study			Checklist of aquatic bugs of Cameroon	
		Genera	Species	Genera	Species^1^ (N)	New report species (Putatively new species)^2^	Genera	Species^3^
Leptopodomorpha	Leptopodidae	0	0	0	0	0 (0)	0	0
	Saldidae	0	0	0	0	0 (0)	0	0
Gerromorpha	Gerridae	5	21	5	9(10)	4 (2)	12	24
Gerromorpha	Hydrometridae	1	5	1	4(4)	3 (2)	1	8
Gerromorpha	Hebridae	1	3	1	1(1)	1 (1)	1	4
Gerromorpha	Macroveliidae	0	0	0	0	0 (0)	0	0
Gerromorpha	Mesoveliidae	1	3	1	2(3)	2 (2)	1	5
Gerromorpha	Veliidae	8	20	2	4(4)	4 (4)	8	24
Gerromorpha	Paraphrynoveliidae	0	0	0	0	0 (0)	0	0
Nepomorpha	Aphelochoridae	1	1	0	0	0 (0)	1	1
Nepomorpha	Belostomatidae	4	6	4	8 (11)	3 (0)	4	9
Nepomorpha	Corixidae	2	3	1	1(1)	0 (0)	2	3
Nepomorpha	Gelastocoridae	0	0	0	0	0 (0)	0	0
Nepomorpha	Helotrephidae	1	1	0	0	0 (0)	1	1
Nepomorpha	Micronectidae	1	3	1	2(2)	2 (2)	1	5
Nepomorpha	Naucoridae	2	6	2	5(5)	1 (0)	4	7
Nepomorpha	Nepidae	3	8	2	8(8)	8 (1)	3	16
Nepomorpha	Notonectidae	4	13	4	10(13)	6 (1)	4	19
Nepomorpha	Ochteridae	1	1	0	0	0 (0)	1	1
Nepomorpha	Pleidae	1	1	0	0	0 (0)	1	1
Nepomorpha	Potamocoridae	0	0	0	0	0 (0)	0	0
	**Total**	**36**	**95**	**24**	**54(62)**	**34 (14)**	**45**	**128**

^1^ Species reported by present study

^2^ Species reported firstly in Cameroun

^3^ Total of aquatic bugs species reported in Cameroon

The number of specimens examined is given in parentheses.

## Discussion

This study complements the work realized by Poisson [3–5] on the diversity of aquatic Heteroptera in Cameroon. In view of the rising worry regarding the potential importance of aquatic bugs in the transmission of the emergent disease Buruli ulcer [6–10], this most up-to-date estimation of aquatic bugs biodiversity is especially relevant. Aquatic bugs biodiversity of Cameroon previously reported by Poisson [3–5, 31–33, 41, 42] reached 15 families including 95 species. With our dataset, we reported in this country 9 new genera and 34 new species records including 14 putative new species. Overall, 45 genera and 125 species of true aquatic and sub-aquatic bugs must be considered in Cameroon (Table 6).

This study underlines the difficulty in identifying the right species based on classical literature in the field without access to museum collections and connection with expert taxonomists. There is a divergence between the number of morphotypes determined *a priori* in the field by non-experts relying on basic identification keys and the morphological analysis carried out by taxonomists from the museum specialized in aquatic bugs. This difference in expertise explains why different morphological species could not be distinguished in the field as different morphotypes and vice versa. In bugs, identification at the species level is difficult in some families, such as Mesoveliidae and Veliidae, and usually requires the presence of males. Some species have different morphological characteristics in males and females for example at *A*. *sardeus* and *E*. *glauca* males and females were initially classified into different morphotypes (Table 1). Moreover, taxonomical keys are lacking to differentiate the nymphal stages. Additional specimens from both sexes and further studies, including possible generic revisions, are needed in order to confirm the status of putative new species.

The use of molecular species delimitation method allows to estimate the specific richness and noticeably, independently of the life stage or the sex of the sample. Thus, molecular identification allows the association of immature stages to adult stages. All nymphal morphotypes could be assigned to an adult morphotypes using this method except for ML2, ML11 and ML17 (Table 5). Even though in most cases there is congruence between the morphological and molecular species, there are some exceptions. In their work on true water bugs, Park *et al*. [17] noted, in some cases, a large unusual intra-specific genetic distance and in some others a very small distance between species.

In our study, in the case of a lack of corresponding molecular species in some morphological species, this could be due to the reduced number of specimens (188) used for the molecular analysis compared to the number of specimens (309) used for the morphological analyses. In other words, the molecular samples might not have been as representative a sample as desired. An explanation for sharing the same molecular putative species between different morphological species is that using a single gene could miss instances of very recent speciation events caused by selection at a few number of loci because a drastic change in morphology could be due to a few genes among which COI is not included. In other words, the “neutral” marker COI might not carry any record of species divergence in that case. Another explanation could be the fact that COI is a mitochondrial gene; it could have introgressed into another species after some hybridization events between the two species. A last hypothesis is that the species based on molecular marker are real but several morphotypes are present within one species.

It is better to complement this type of study with phylogenetic analyses to determine relationships between species or group of species, but due to the limited sampling size of our study and the use of a single mitochondrial gene that is not intended for building phylogeny, we performed only the separation of the molecular species within the families of aquatic bugs identified. Previous works based on many loci described the phylogenetic relationships of the aquatic bugs more accurately. Hebsgaard *et al*. [43] used 16S, 28S and COI/COII, whereas Hua *et al*. [44] used 37 mitochondrial loci to obtain a well-resolved phylogeny of the true aquatic bugs. But these studies concerned Europe, USA, Australia, Philippines, Madagascar and Vietnam whereas the present study is the first in the Afrotropical region.

## Conclusion

This study improves our knowledge on the diversity and distribution of aquatic bugs in Cameroon and confirms that COI can reliably be used to identify species in most families of aquatic bugs described here apart from the few exceptions observed. In the near future, molecular identification could also help to routinely identify aquatic bug species of importance in the transmission of the bacillus causing Buruli ulcer in human in the tropical region.

This pioneering study will be extended to other Afrotropical region to better document the biodiversity of aquatic bugs in this part of the world.

## Supporting Information

S1 Fig Molecular putative species delimited by ABGD according to aquatic bug families using partition 1 in Nepomorpha infra-order. Each color represents one ABGD putative specie delimited with associated nymph corresponding to code situated in front of vertical line respectively families’ initial name followed by putative specie number and ML followed by putative nymph specie number, before vertical line Mor = adult morphotype code following by morphotype number and individual number, ML = nymph morphotype code following by morphotype number and individual number.
(TIFF)

S2 Fig Molecular putative species delimited by ABGD according to aquatic bug families using partition 1 in Gerromorpha infra-order. Each color represents one ABGD putative specie delimited with associated nymph corresponding to code situated in front of vertical line respectively families’ initial name followed by putative specie number and ML followed by putative nymph specie number, before vertical line Mor = adult morphotype code following by morphotype number and individual number, ML = nymph morphotype code following by morphotype number and individual number.
(TIFF)

S1 File Fasta file of sequences data set underlying the findings in our study in the manuscript with one outgroup sequence. Sequence name corresponds to families initial name followed by individual code number.
(FAS)
