# Supplementary figures and images for: Molecular Species Delimitation and Morphology of Aquatic and Sub-Aquatic Bugs (Heteroptera) in Cameroon
Source: PLoS One. 2016 May 5;11(5):e0154905. doi: 10.1371/journal.pone.0154905 (PMC4858268; doi:10.1371/journal.pone.0154905)

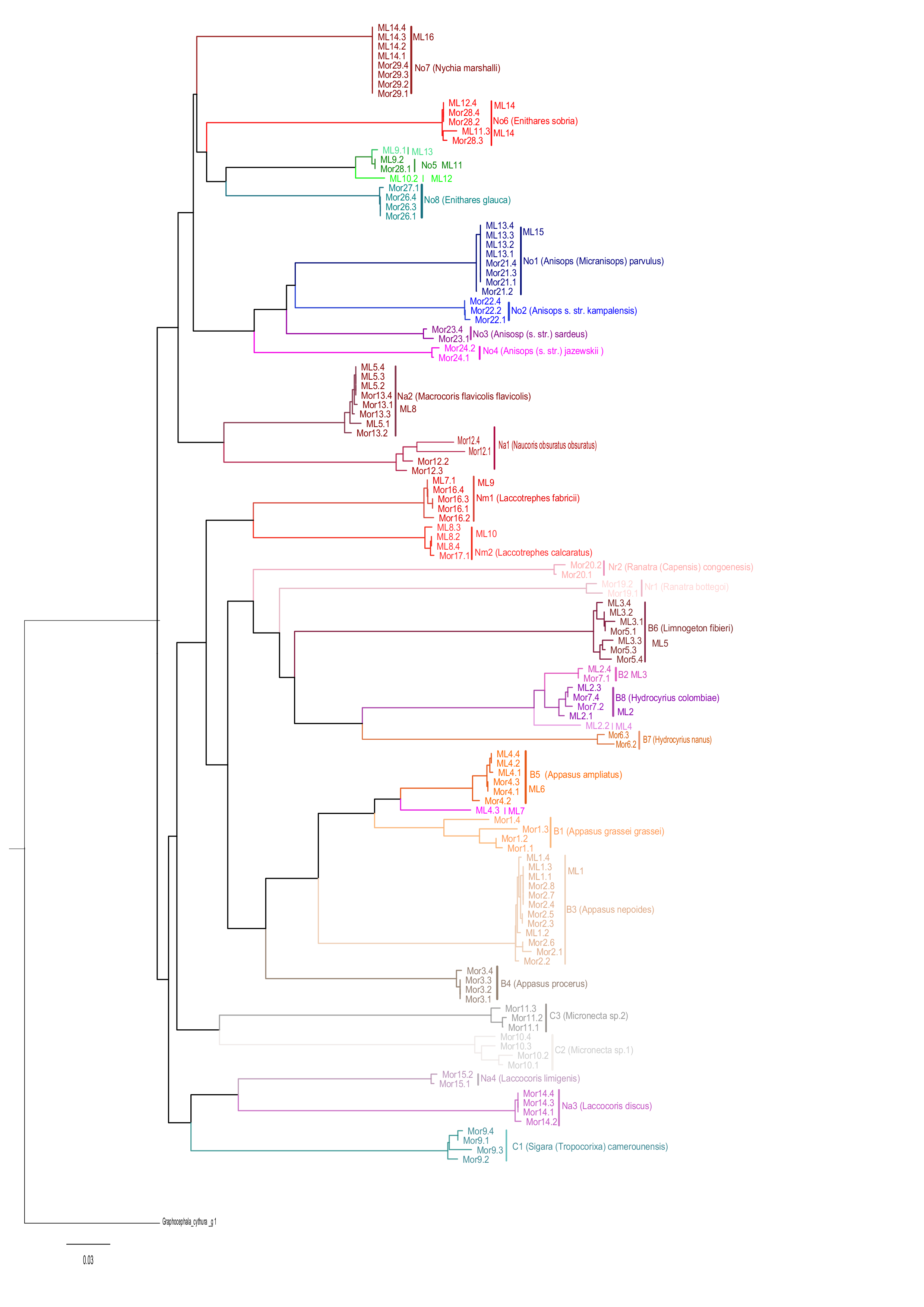

Supplement: S1 Fig — Each color represents one ABGD putative specie delimited with associated nymph corresponding to code situated in front of vertical line respectively families’ initial name followed by putative specie number and ML followed by putative nymph specie number, before vertical line Mor = adult morphotype code following by morphotype number and individual number, ML = nymph morphotype code following by morphotype number and individual number. (TIFF) [file pone.0154905.s001.tiff]

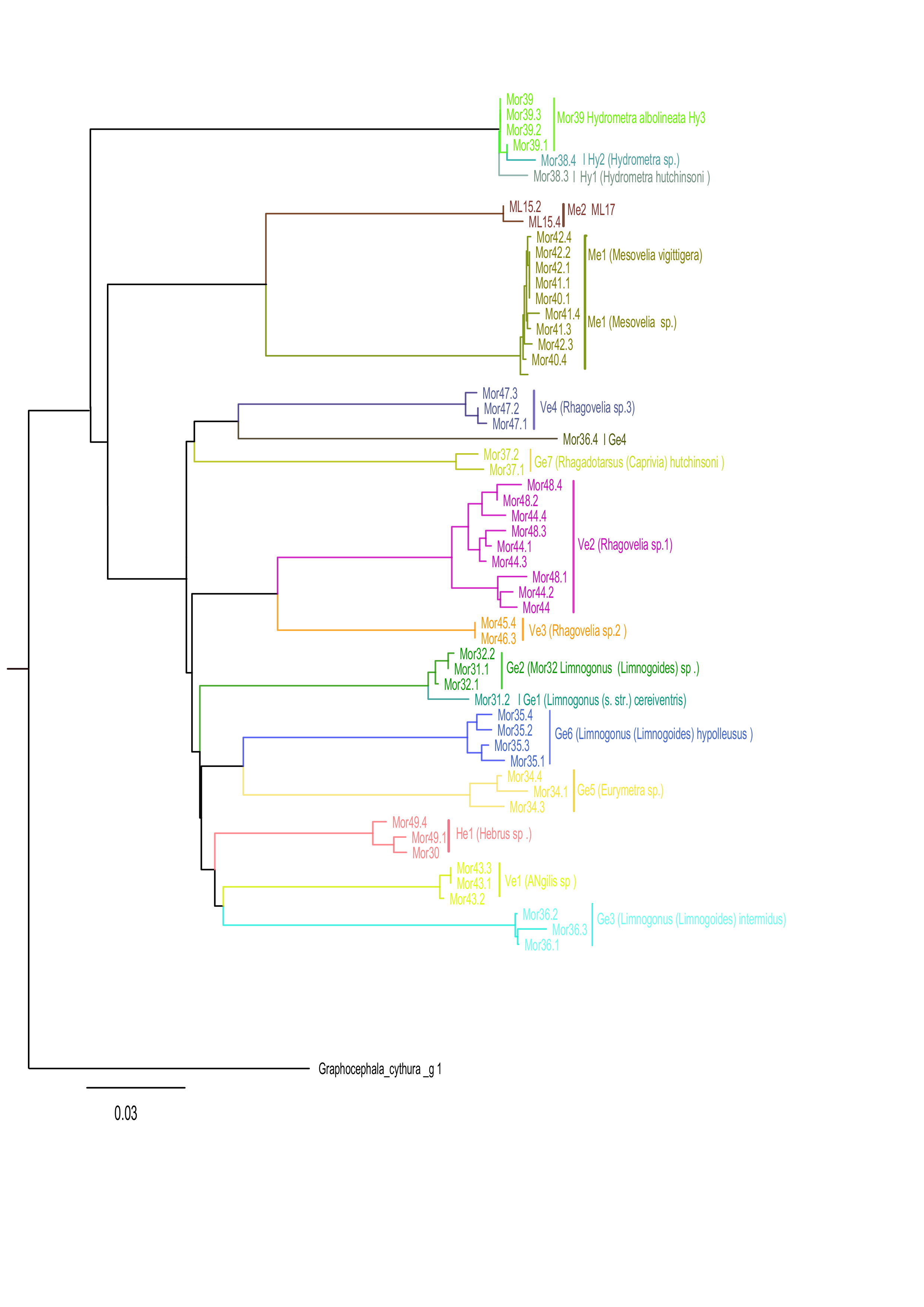

Supplement: S2 Fig — Each color represents one ABGD putative specie delimited with associated nymph corresponding to code situated in front of vertical line respectively families’ initial name followed by putative specie number and ML followed by putative nymph specie number, before vertical line Mor = adult morphotype code following by morphotype number and individual number, ML = nymph morphotype code following by morphotype number and individual number. (TIFF) [file pone.0154905.s002.tiff]
